# Usefulness of video-laryngoscopy with the Airway Scope for intubation performance and learning: an experimental manikin controlled study

**DOI:** 10.1186/s13613-016-0182-0

**Published:** 2016-08-26

**Authors:** Pierre-Louis Declercq, Michael Bubenheim, Stéphanie Gelinotte, Kévin Guernon, Jean-Baptiste Michot, Vincent Royon, Dorothée Carpentier, Gaëtan Béduneau, Fabienne Tamion, Christophe Girault

**Affiliations:** 1 Department of Medical Intensive Care, Rouen University Hospital, Rouen Cedex, France; 2 Department of Clinical Research Support, Biostatistics Unit, Rouen University Hospital, Rouen Cedex, France; 3 UPRES EA 3830-IRIB, Institute for Biomedical Research, Rouen University, Rouen Cedex, France; 4 Service de Réanimation Médicale, Hôpital Charles Nicolle, Centre Hospitalier Universitaire-Hôpitaux de Rouen, 1, Rue de Germont, 76031 Rouen Cedex, France

**Keywords:** Intubation, Video-laryngoscopy, Airway Scope, Macintosh, Simulation, Learning

## Abstract

**Background:**

Different video-laryngoscopes (VDLs) for endotracheal intubation (ETI) have recently been developed. We compared the performance of the VDL Airway Scope (AWS) with the direct laryngoscopy by Macintosh (DLM) for ETI success, time and learning.

**Methods:**

We performed an experimental manikin controlled study. Twenty experienced (experts) and 40 inexperienced operators (novices) for DLM-ETI were enrolled. None of them had experience with the use of AWS-VDL. Novices were assigned to start learning with DLM or AWS, and two sub-groups of 20 novices were formed. Experts group constituted the control group. Each participant performed 10 ETI attempts with each device on the same standard manikin. The primary endpoint was the ETI success probability. Secondary endpoints were ETI time, technical validity and qualitative evaluation for each technique. We also assessed the learning order and the successive attempts effects for these parameters.

**Results:**

Overall, 1200 ETI attempts were performed. ETI success probability was higher with the AWS than with the DLM for all operators (98 vs. 81 %; *p* < 0.0001) and for experts compared to novices using devices in the same order (97 vs. 83 %; *p* = 0.0002). Overall ETI time was shorter with the AWS than with the DLM (13 vs. 20 s; *p* < 0.0001) and for experts compared to novices using devices in the same order (11 vs. 21 s; *p* < 0.0001). Among novices, those starting learning with AWS had higher ETI success probability (89 vs. 83 %; *p* = 0.03) and shorter ETI time (18 vs. 21 s; *p* = 0.02). Technical validity was found better with the AWS than DLM for all operators. Novices expressed global satisfaction and device preference for the AWS, whereas experts were indifferent.

**Conclusions:**

AWS-VDL permits faster, easier and more reliable ETI compared to the DLM whatever the previous airway ETI experience and could be a useful device for DLM-ETI learning.

## Background

Endotracheal intubation (ETI) is a routine life-saving procedure for airway management, and direct laryngoscopy with the Macintosh laryngoscope (DLM) is probably the most common way to perform ETI. Nevertheless, ETI using DLM (DLM-ETI) is a high-risk procedure, which can lead to vital complications, particularly in intensive care unit (ICU) patients [1, 2]. These risks are related to the critical setting as well as the underlying disease (hypoxemia, hemodynamic instability) and technical conditions of ETI [3]. Furthermore, learning and regular training are needed to sufficiently acquire skills and experience with the DLM technique [4–7].

In the past few years, new devices called video-laryngoscopes (VDLs) have been developed for improving airway management. With optical or video technique, most of these instruments allow to indirectly visualize the larynx and control the endotracheal tube (ETT) passing throughout the glottis [8]. To date, these different VDLs have been mainly used in the operating and emergency room or prehospital setting [7, 8]. Although VDL has been more recently applied in ICU setting [9–16], some authors have shown that ETI with VDL (VDL-ETI) could be associated with longer ETI time, lower oxygen saturation and even higher mortality as compared to conventional DLM-ETI [9, 13]. In fact, due to their technical conception, VDL may have their respective advantages and pitfalls, and their place may be still controversial in the ICU where airway management appears more hazardous and insufficiently studied [17].

**Fig. 1:**
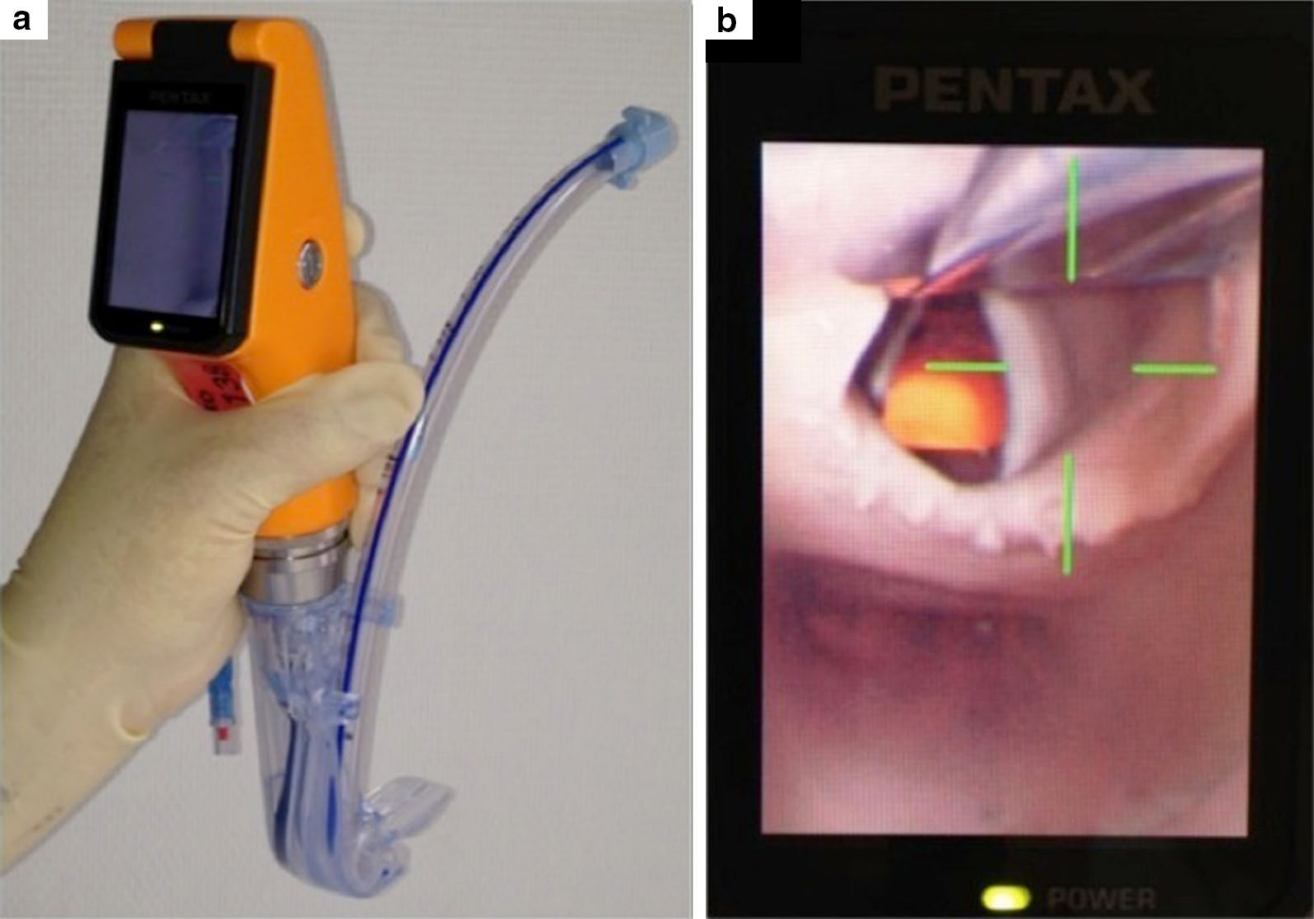
Airway Scope® (AWS; Pentax Corp., Tokyo, Japan) device. **a** The AWS is a portable and battery-operated video-laryngoscope (VDL) with an integrated and wide-viewing-angle (180°) liquid crystal display (LCD) monitor (6.1 cm) providing an indirect laryngoscopy of the airway via a charged coupled device attached to the blade tip of the AWS. The single-use intlock blade has a specific tube guide to accept the ETT (internal diameter between 6.5 and 8 mm). AWS-ETI requires to load and lift the epiglottis with the AWS blade tip. **b** Once the target signal on the LCD monitor is aligned with the glottis opening, the ETT is passed through the vocal cords (**a**). Then, the AWS is removed laterally, leaving the ETT in place

Therefore, there is a need to rigorously assess each VDL device on an experimental and clinical basis in order to optimize ETI success. The Airway Scope (AWS^®^, Pentax Corp., Tokyo, Japan) is a new VDL device first reported in 2006 (Fig. 1) [18]. We hypothesized that ETI success rate, time and learning to perform a reliable ETI could be optimized by the use of the AWS as compared to the DLM, in experienced and inexperienced operators.

## Methods

We conducted an experimental manikin controlled study to compare the AWS and DLM performance for ETI success and time, as well as ETI learning between experienced and inexperienced operators. All operators agreed to participate in this manikin study, and as it was a teaching study, which enrolled no patients and did not evaluate physiopathological process, our local ethics committee (Comité de Protection des Personnes du Nord Ouest I) stated that no ethical approval and no consent were required (conclusion date February 20, 2015, chairperson address: Pr F. Bauer, Hôpital Charles Nicole, Centre Hospitalier-Universitaire de Rouen, 1 rue de Germont 76031, France). No company sponsored the study nor supplied any device used. Experienced operators, named “experts,” were 20 senior ICU physicians (*n* = 9) or anesthesia residents (*n* = 11) who had previously performed more than 100 DLM-ETI but with no prior experience with AWS-ETI. Inexperienced operators, named “novices,” were 40 medical students with no prior experience with either of the two ETI techniques. Male and female participants were equally distributed for each novice sub-group (10 vs. 10), except for the expert sub-group (13 vs. 7, respectively).

ETI was performed on a simple manikin head (Cparlene^®^, Adult Airway Larry manikin, CPR Savers & First Aid Supply, Scottsdale, USA) without any difficult ETI criteria, either with the DLM (blade size no. 3) or with the AWS. The AWS is a portable and battery-operated VDL with an integrated and wide-viewing-angle (180°) liquid crystal display (LCD) monitor (6.1 cm) providing an indirect laryngoscopy of the airway via a charged coupled device attached to the blade tip of the AWS. The single-use intlock blade has a specific tube guide to accept the ETT (Fig. 1a). Concerning AWS-ETI, in contrast to DLM-ETI, the ETT must be preloaded on the tube guide and ETI requires to load and lift the epiglottis with the blade tip. Once the target signal on the LCD monitor is aligned with the glottis opening, the ETT is passed through the vocal cords (Fig. 1b). Then, the AWS is removed laterally, leaving the ETT in place. For AWS-ETI, the ETT was already preload in the blade-side channel before starting the ETI attempt. All ETI were performed with no introducer and a lubricated low-pressure cuffed ETT (Portex^®^, Smiths Medical, St Paul, USA) with an internal diameter of 7.5 mm. The manikin head was maintained in a neutral position, i.e., not in improved Jackson’s position. The good ETT position was confirmed by lung inflation with a manual self-inflating bag (AMBU^®^ Mark IV, Ambu Corp., Ballerup, Denmark).

All inexperienced students underwent 30-min theoretical and practical training, including an ETI demonstration with each device by one experienced investigator in both techniques. During this training, students had only one attempt on the manikin with each device. The same program was given to all experts, for the AWS alone. Then, each participant performed 10 consecutive ETI attempts on the manikin with both devices. The novice operators were assigned to two different study sub-groups according to the device order of the 10 attempts, whereas the experts group performed all attempts in the same order. Therefore, three sub-groups of 20 operators were organized according to their ETI experience and device order, i.e., expert (DLM then AWS), novice 1 (DLM then AWS) and novice 2 (AWS then DLM) sub-groups.

The primary endpoint of the study was the ETI success probability with each technique. ETI success was defined as an ETI performed within 60 s, with ETT in place in the trachea. Consequently, ETI failure could have been either due to a delayed ETI (>60 s) or due to esophageal intubation. Several secondary endpoints were assessed. The ETI time was defined as the time taken from the blade (DLM or AWS) first passing the incisors until ETT passage through the vocal cords. We also reported the time between the ETT passage through the vocal cords and lung inflation. The ETI technical validity for each attempt with both devices was scored and visually assessed by collecting the following adverse events: for the DLM: dental trauma based on the excessive pressure exerted by the blade on the upper incisors, epiglottis loading and default of epiglottis traction due to a lack of handle traction up and forward; for the AWS: ETT dislodgment (mobilization outside the blade channel or ablation when removing the AWS laterally), epiglottis not loaded and epiglottis luxation as well as blade malposition in the glottis. The number of esophageal intubations for both devices and the Cormack and Lehane grade for the DLM were also recorded [19].

After performing 10 ETI attempts with each device, all participants were asked to rate the following qualitative evaluation criteria using a 5-point Likert scale: ease of assembly of the device, difficulty with ETT manipulation, force of traction required for ETI, as well as the global satisfaction with the technique. It was also asked for their preference regarding the techniques for everyday use.

### Statistical analysis

Given the dependence between successive attempts of the same volunteer, a mixed-effect logistic regression model was used [20]. As operator’s selection was based on a block design, sex was treated as a random block effect. Because observed times appeared skewed, a mixed-effect regression model with auto-correlated errors was used with time being lognormal. Point and interval estimates were then retransformed using the exponential function to present the results in conventional units. Since no failure was observed in all operator groups at several occasions, the *p* value for differences between attempts using AWS was derived by comparing the results of each attempt with respect to the first one, the one with the lowest number of successes for this device, and then correcting for multiple testing using Holm’s procedure. In case of undefined logits, the sign test was carried out for local testing and simple logistic regression was used in all other cases.

As regards technical events, the number per operator was described using median, first and third quartiles [*Q*1–*Q*3]. In order to assess whether operator groups differed with respect to the occurrence of these events, Freeman–Halton’s test was used when results were heavily tied, i.e., events happened to less than 8 operators, otherwise the Kruskal–Wallis test was taken. In order to come to know whether operators assessed both devices differently, the sign test was used.

A *p* value less than 0.05 was considered statistically significant.

## Results

Sixty participants performed 20 ETI attempts each, yielding 400 ETI attempts for each sub-group, i.e., a total of 1200 ETI attempts were assessed.

 Considering all ETI attempts, we observed 111 failures with the DLM and 14 with the AWS (Table 1). In univariable analysis, the overall ETI success probability was significantly higher for the AWS compared to the DLM (98 vs. 81 %; *p* < 0.0001), for experts compared to novices 1 (97 vs. 83 %; *p* = 0.0002) and for novices 2 compared to novices 1 (89 vs. 83 %; *p* = 0.03). No significant learning effect of successive attempts was observed for ETI success. These findings were confirmed by multivariable analysis with independent factors (Table 1). As regards univariable analysis, ETI time was significantly shorter with AWS compared to DLM for all operators (13 vs. 20 s; *p* < 0.0001), for experts compared to novices 1 (11 vs. 21 s; *p* < 0.0001) and for novices 2 compared to novices 1 (18 vs. 21 s; *p* = 0.02) (Table 2). When compared to the first attempt, ETI time was significantly reduced at the third attempt (21 vs. 17 s; *p* = 0.003) and for all further attempts (Table 2). Multivariable analysis with independent factors led to the same conclusions (Table 2).

**Table 1:** Factors potentially influencing endotracheal intubation success probability

	All attempts (*n* = 1200)		Univariable analysis*					Multivariable analysis***				
			Success** probability	95 % CI limits				Success probability for novices 1	95 % CI limits			
	Number of attempts, *n*	Failure attempts, *n* (%)		Lower	Upper	Odds ratio	*p* value		Odds ratio	Lower	Upper	*p* value
ETI device	1200	125 (10.4)										
DLM	600	111 (18.5)	0.81	0.67	0.90	1.00	<0.0001	0.69	–	0.005	1.00	<0.0001
AWS	600	14 (2.3)	0.98	0.94	0.99	9.57		0.96	–	0.09	1.00	
Operator sub-group (device order)												
Experts (DLM then AWS)	400	10 (2.5)	0.97	0.94	0.99	8.04	0.0002	–	9.06	4.17	19.71	<0.0001
Novices 1 (DLM then AWS)	400	70 (17.5)	0.83	0.72	0.90	1.00	–	–	1.00	–	–	–
Novices 2 (AWS then DLM)	400	45 (11.3)	0.89	0.81	0.94	1.68	0.03	–	1.80	1.12	2.89	0.02
Attempt number in the series of 10 attempts												
1	120	17 (14.2)	0.87	0.73	0.94	1.00	Reference	–	–	–	–	–
2	120	18 (15)	0.85	0.71	0.93	0.89	0.70	–	–	–	–	–
3	120	12 (10)	0.90	0.79	0.96	1.41	0.39	–	–	–	–	–
4	120	17 (14.2)	0.86	0.72	0.93	0.95	0.88	–	–	–	–	–
5	120	8 (6.7)	0.93	0.84	0.97	2.21	0.08	–	–	–	–	–
6	120	13 (10.8)	0.89	0.77	0.95	1.29	0.52	–	–	–	–	–
7	120	11 (9.2)	0.91	0.80	0.96	1.56	0.28	–	–	–	–	–
8	120	8 (6.7)	0.93	0.84	0.97	2.21	0.08	–	–	–	–	–
9	120	10 (8.3)	0.92	0.82	0.97	1.73	0.19	–	–	–	–	–
10	120	11 (9.2)	0.90	0.79	0.96	1.42	0.35	–	–	–	–	–

*ETI* endotracheal intubation, *DLM* direct laryngoscopy with Macintosh, *AWS* video-laryngoscopy with Airway Scope®

* Univariable analysis, all of the 1200 attempts have been analyzed separately according to 3 variables: ETI device (DLM vs. AWS), operator sub-group (experts vs. novices 1 and novices 1 vs. novices 2) and the attempt number in the series of 10 attempts

** Probability of a single successful attempt for one operator

*** Novices 1 is the reference sub-group of the multivariable analysis, i.e., the simultaneous analysis of 2 variables: ETI device (DLM vs. AWS) and operator sub-group (experts vs. novices 1 and novices 1 vs. novices 2). For example, the probability of a successful attempt is 9.06 higher for an expert than a novice 1

**Table 2:** Factors potentially influencing endotracheal intubation time

	All attempts (*n* = 1200)		Univariable analysis*				Multivariable analysis**				
			ETI Time (s)	95 % CI limits		*p* value	ETI time at first attempt for novices 1 (s)	Time multiplier***	95 % CI limits		*p* value
	Number of attempts, *n*	Sample arithmetic mean time (s)		Lower	Upper				Lower	Upper	
ETI device	1200	19.63									
DLM	600	24.52	20.44	16.18	25.83	<0.0001	35.65	–	29.61	42.93	<0.0001
AWS	600	14.73	12.72	10.08	16.06		22.51	–	18.75	27.02	
Operator sub-group (device order)											
Experts (DLM then AWS)	400	12.53	11.01	9.28	13.07	<0.0001	–	0.54	0.49	0.58	<0.0001
Novices 1 (DLM then AWS)	400	25.35	20.51	17.29	24.32		–	1.00	–	–	–
Novices 2 (AWS then DLM)	400	21.00	18.37	15.49	21.79	0.02	–	0.83	0.77	0.90	<0.0001
Attempt number in the series of 10 attempts											
1	120	24.92	20.99	16.42	26.84	Reference	–	1.00	–	–	–
2	120	23.63	19.44	15.16	24.92	0.16	–	0.91	0.83	1.01	0.08
3	120	20.62	17.09	13.33	21.93	0.003	–	0.80	0.71	0.89	0.0001
4	120	19.56	15.92	12.41	20.42	<0.0001	–	0.74	0.66	0.84	<0.0001
5	120	18.76	15.22	11.87	19.51	<0.0001	–	0.71	0.63	0.80	<0.0001
6	120	18.22	14.87	11.59	19.09	<0.0001	–	0.69	0.62	0.77	<0.0001
7	120	18.65	15.34	11.94	19.72	<0.0001	–	0.71	0.63	0.80	<0.0001
8	120	17.81	14.59	11.35	18.75	<0.0001	–	0.68	0.60	0.76	<0.0001
9	120	17.03	14.06	10.96	18.03	<0.0001	–	0.65	0.58	0.73	<0.0001
10	120	17.06	13.69	10.61	17.66	<0.0001	–	0.63	0.57	0.70	<0.0001

*ETI* endotracheal intubation, *DLM* direct laryngoscopy with Macintosh, *AWS* video-laryngoscopy with Airway Scope®

* Univariable analysis, all the 1200 attempts have been analyzed separately according to 3 variables: ETI device (DLM vs. AWS), operator sub-group (experts vs. novices 1 and novices 1 vs. novices 2) and the attempt number in the series of 10 attempts

** Novices 1 at the first attempt serve as reference situation of the multivariable analysis, i.e., the simultaneous analysis of 3 variables: ETI device (DLM vs. AWS), operator sub-group (experts vs. novices 1 and novices 1 vs. novices 2) and the attempt number in the series of 10 attempts

*** Multiplicative factor allowing to estimate ETI time from the novices 1 ETI time for the others situations. For example, ETI time for an expert using AWS at first attempt is estimated to be 12.16 s, i.e., 22.51 × 0.54 × 1

All experts succeeded their 10 AWS-ETI attempts, whereas 9 novices failed at least once (3 and 6 novices in sub-groups 1 and 2, respectively). Fourteen experts (70 %) succeeded all DLM-ETI attempts in contrast to 3 (15 %) and 7 (35 %) novices in sub-groups 1 and 2, respectively (data not shown).

**Fig. 2:**
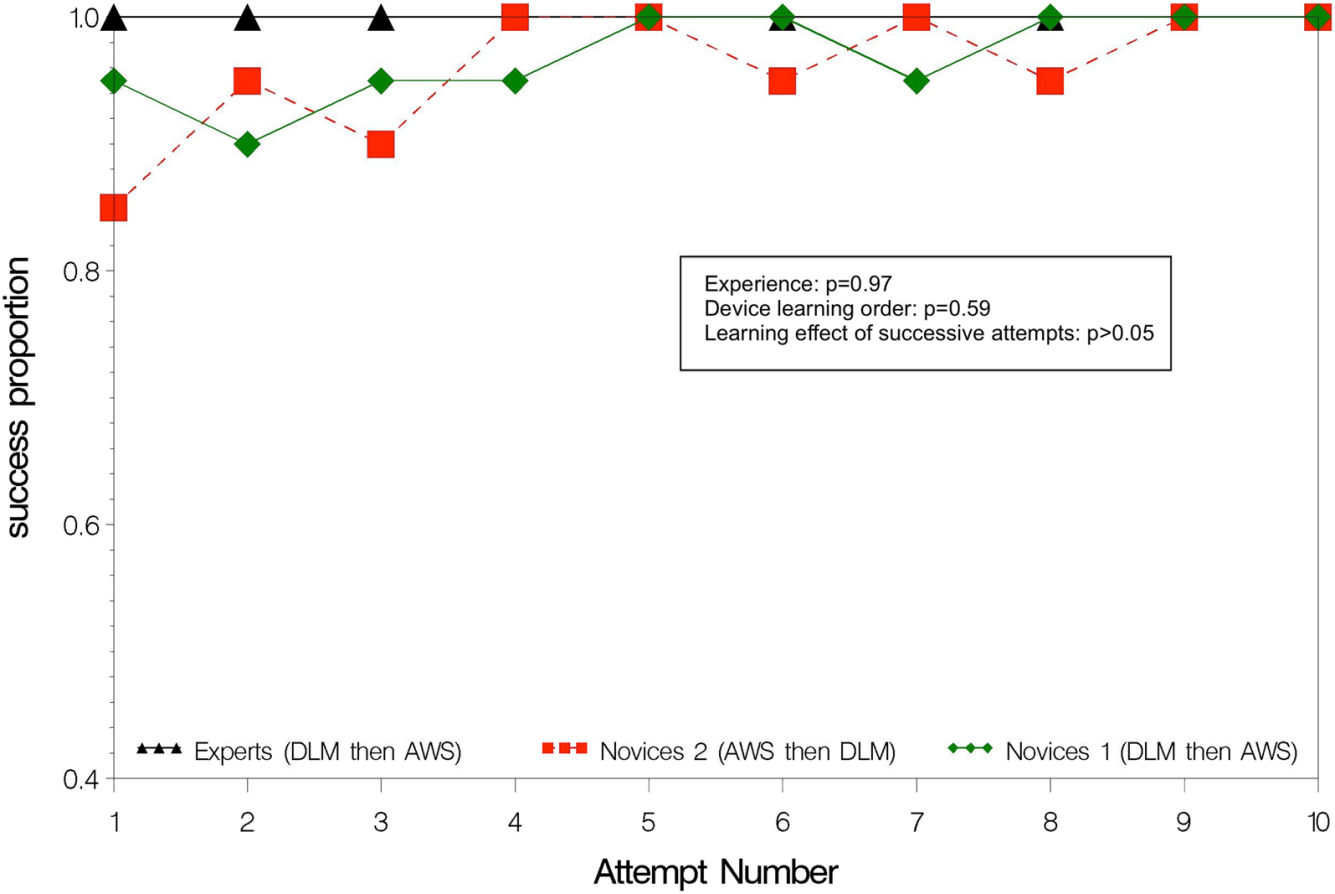
AWS-ETI learning curves according to ETI success rate by attempt and operator sub-group. *ETI* endotracheal intubation, *DLM* direct laryngoscopy with Macintosh, *AWS* video-laryngoscopy with Airway Scope^®^. Success proportion is the observed number of successes among 100 attempts for the 3 study sub-groups (experts, novices 1 and novices 2) according to the attempt number in a series of ten attempts and the device used (DLM or AWS). *p* values refer to homogeneity tests for experience (experts compared to novices 1), device learning order (novices 1 compared to novices 2), and to the global test for any difference between attempts, which evaluate the learning effect of successive attempts

**Fig. 3:**
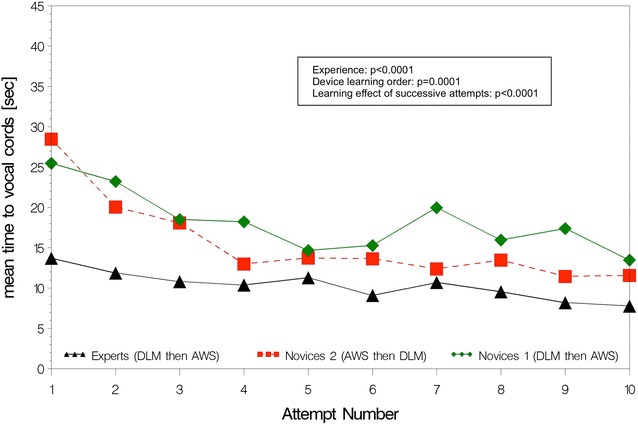
AWS-ETI learning curves according to ETI time by attempt and operator sub-group. *ETI* endotracheal intubation, *DLM* direct laryngoscopy with Macintosh, *AWS* video-laryngoscopy with Airway Scope^®^. Mean time to vocal cords corresponds to ETI time (time taken from the blade (DLM or AWS) first passing the incisors until ETT passage through the vocal cords) in seconds according to the attempt number in a series of ten attempts and the device used (DLM or AWS). *p* values refer to homogeneity tests for experience (experts compared to novices 1), device learning order (novices 1 compared to novices 2), and to the global test for any difference between attempts, which evaluate the learning effect of successive attempts

**Fig. 4:**
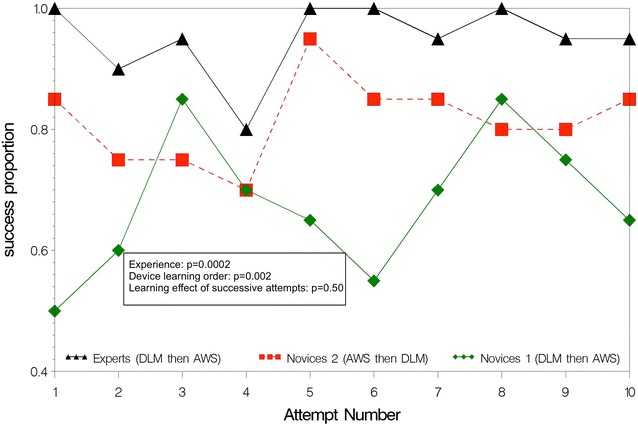
DLM-ETI learning curves according to ETI success rate by attempt and operator sub-group. *ETI* endotracheal intubation, *DLM* direct laryngoscopy with Macintosh, *AWS* video-laryngoscopy with Airway Scope^®^. Success proportion is the observed number of successes among 100 attempts for the 3 study sub-groups (experts, novices 1 and novices 2) according to the attempt number in a series of ten attempts and the device used (DLM or AWS). *p* values refer to homogeneity tests for experience (experts compared to novices 1), device learning order (novices 1 compared to novices 2), and to the global test for any difference between attempts, which evaluate the learning effect of successive attempts

**Fig. 5:**
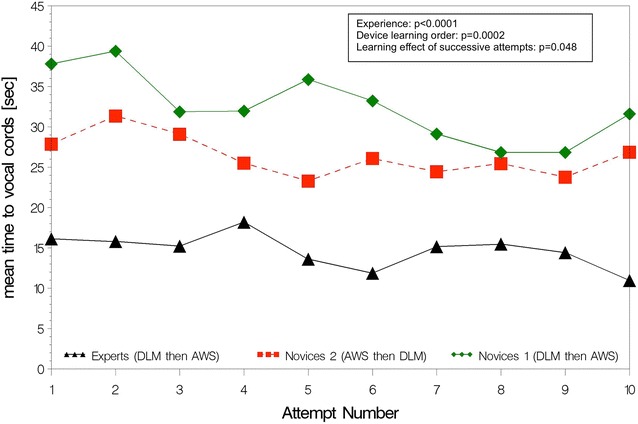
DLM-ETI learning curves according to ETI time by attempt and operator sub-group. *ETI* endotracheal intubation; *DLM* direct laryngoscopy with Macintosh, *AWS* video-laryngoscopy with Airway Scope®. Mean time to vocal cords corresponds to ETI time (time taken from the blade (DLM or AWS) first passing the incisors until ETT passage through the vocal cords) in seconds according to the attempt number in a series of ten attempts and the device used (DLM or AWS). *p* values refer to homogeneity tests for experience (experts compared to novices 1), device learning order (novices 1 compared to novices 2), and to the global test for any difference between attempts, which evaluate the learning effect of successive attempts

 Empirical ETI learning curves are shown separately for AWS (Figs. 2, 3) and DLM (Figs. 4, 5). The AWS-ETI success proportion curves for novices are close to that of experts (Fig. 2). Looking at AWS alone and noting that no failure was observed for several attempts, no evidence was found for an effect of experience, learning order and successive attempts (Fig. 2). ETI time for AWS alone was significantly shorter for experts when compared to novices 1 (9 vs. 16 s; *p* < 0.0001), when learning order began with AWS instead of DLM (13 vs. 16 s; *p* = 0.0001) and with increasing number of attempts (10 and 19 s, at first and tenth attempts, respectively; *p* < 0.0001) (Fig. 3). Learning curves were different for each sub-group with DLM (Figs. 4, 5). When considering DLM alone, DLM-ETI success proportion was significantly higher for experienced operators as compared to novices 1 (95 vs. 82 %; *p* = 0.0002), when learning started with AWS as compared to DLM (82 vs. 68 %; *p* = 0.002), but no significant effect of successive attempt was found (Fig. 4). ETI time for DLM alone changed significantly between the first and the tenth attempts (24 and 18 s, respectively; *p* = 0.048) and was shorter for experts (13 vs. 28 s; *p* < 0.0001) and for novices 2 (23 vs. 28 s; *p* = 0.0002) as compared to novices 1 (Fig. 5).

In univariable analysis, the time between the ETT passage through the vocal cords and lung inflation was found significantly shorter for all operators when the DLM was used as compared to AWS (9 vs. 11 s; *p* < 0.0001), when ETI was performed by experts (7 vs. 13 s; *p* < 0.0001) or by novices 2 (12 vs. 13 s; *p* = 0.005) as compared to novices 1, and with increasing number of attempts (12 and 9 s, at first and tenth attempts, respectively; *p* < 0.001). All these findings were confirmed by multivariable analysis (data not shown).

ETI technical validity and adverse events are reported in Tables 3 and 4. The DLM-ETI technical validity appeared higher for experts than for both novice sub-groups (*p* = 0.0005; Table 3). All adverse events occurred more frequently in these two sub-groups, and dental pressure was the main event reported. There were few adverse events with the AWS-ETI and virtually no difference in technical validity between the three sub-groups (Table 4). The main events reported were epiglottis luxation for experts (*p* = 0.04) and delayed ETI time for both novice sub-groups (*p* = 0.04).

**Table 3:** Technical validity and adverse events for endotracheal intubation with the DLM by operator sub-group

	Operator sub-group (device order)						*p* value*
	Experts (DLM then AWS) *n* = 20		Novices 1 (DLM then AWS) *n* = 20		Novices 2 (AWS then DLM) *n* = 20		
	Events ** (out of 200 attempts)	Median [*Q*1–*Q*3]***	Events** (out of 200 attempts)	Median [*Q*1–*Q*3]***	Events ** (out of 200 attempts)	Median [*Q*1–*Q*3]***	
Technically valid attempts, *n*	146	9 [7–9]	61	2 [1–4]	84	4 [2–6]	0.0005
Adverse technical events							
Esophageal intubation, *n*	6	0 [0–0]	34	1 [0–2]	21	0 [0–2]	0.02
Delayed ETI time (>60 s), *n*	4	0 [0–0]	30	1 [0–2]	16	0 [0–1]	0.003
Dental pressure, *n*	49	1 [0–3]	78	4 [2–5]	76	3 [2–5]	0.02
Epiglottis loading, *n*	1	0 [0–0]	26	1 [0–2]	25	1 [0–2]	0.0001
Lack of traction, *n*	3	0 [0–0]	35	1 [0–4]	15	0 [0–1]	0.002
Cormack–Lehane grade (median)	–	1 [1, 2]	–	2 [1, 2]	–	2 [1, 2]	0.008

*ETI* endotracheal intubation, *DLM* direct laryngoscopy with Macintosh, *AWS* video-laryngoscopy with Airway Scope®

* Kruskal–Wallis test

** Total number of each event on 200 attempts in each sub-group with DLM

*** Median [1st–3rd quartile] per operator of the number of events on 10 attempts with DLM in each sub-group

**Table 4:** Technical validity and adverse events for endotracheal intubation with the AWS by operator sub-group

	Operator sub-group (device order)						*p* value
	Experts (DLM then AWS) *n* = 20		Novices 1 (DLM then AWS) *n* = 20		Novices 2 (AWS then DLM) *n* = 20		
	Events* (out of 200 attempts)	Median [*Q*1–*Q*3]**	Events* (out of 200 attempts)	Median [*Q*1–*Q*3]**	Events* (out of 200 attempts)	Median [*Q*1–*Q*3]**	
Technically valid attempts, *n*	179	9 [8–10]	177	9 [9, 10]	184	9 [9, 10]	0.59****
Adverse technical event							
Esophageal intubation, *n*	0	0 [0–0]	1	0 [0–0]	1	0 [0–0]	1.0***
Delayed ETI time (>60 s.), *n*	0	0 [0–0]	5	0 [0–0]	7	0 [0–0]	0.04***
Epiglottis luxation, *n*	21	1 [0–2]	13	0 [0–1]	8	0 [0–1]	0.04****
Epiglottis unloaded, *n*	0	0 [0–0]	2	0 [0–0]	0	0 [0–0]	0.32***
ETT dislodgment, *n*	0	0 [0–0]	0	0 [0–0]	3	0 [0–0]	0.1***
Blade malposition in glottis, *n*	0	0 [0–0]	9	0 [0–0]	5	0 [0–0]	0.43***

*ETI* endotracheal intubation, *DLM* direct laryngoscopy with Macintosh, *AWS* video-laryngoscopy with Airway Scope®

* Total number of each event on 200 attempts in each sub-group with AWS

** Median [1st–3rd quartile] per operator of the number of events on 10 attempts with AWS in each sub-group

*** Freeman–Halton’s test

**** Kruskal–Wallis test

Concerning qualitative assessment of ETI technique (data not shown), DLM was considered easier to assemble than the AWS by expert and novice 2 sub-groups. ETT manipulation with DLM was considered easier by experts although novices reported no difference. All sub-groups agreed that DLM-ETI required more force than did AWS-ETI. ETI global satisfaction and preference were in favor of the AWS in both novice sub-groups, whereas experts were indifferent.

## Discussion

To our knowledge, this is the largest controlled manikin study comparing a new device, AWS-VDL, with the DLM technique for ETI. The AWS allowed faster, easier and more reliable ETI performance than the DLM whatever the previous airway ETI experience. Interestingly, novice operators who started with the AWS performed DLM-ETI more efficiently than those starting with the DLM, which suggests AWS to be a useful device for DLM-ETI learning. Furthermore, there was no effect of successive attempts on ETI success probability with both devices. This could mean that for novice operators, AWS-ETI learning on manikin is achieved after the first attempts. By contrast, and as demonstrated in previous studies [4–6], 10 ETI attempts were found insufficient for novice operators to perform DLM-ETI with reliably. Finally, our results demonstrate that AWS-ETI acquisition may require less operator skill than the DLM.

Our results for AWS-ETI performance are in agreement with those of one previous manikin study comparing the AWS with DLM in 31 inexperienced nurses [21]. Two prospective randomized clinical studies reported that the AWS allowed to decrease ETI time and failure by inexperienced residents as compared to DLM [22, 23]. In routine anesthesia, the AWS was also able to significantly improve the laryngeal view being more reliable in case of unanticipated difficult ETI as compared to DLM [24].

The time between the ETT passage through the vocal cords and lung inflation was found shorter with the DLM for all operators. The device itself could explain this finding in part. Indeed, the DLM can be more easily removed than the AWS, since the operator has to take care not to accidentally remove the tube inserted in the AWS side channel (Fig. 1a) while he removes the AWS blade laterally [18]. This adverse event occurred three times in six hundred ETI attempts with AWS in our study. In addition, the time required to remove the AWS from the mouth should not delay the lung inflation. In practice, it is possible to ventilate the more hypoxemic patients manually before removing the AWS device. The time between ETI and lung inflation was also found shorter for experts compared to novices. This could be explained by the experts’ greater experience to manage cuff inflation as well as manual self-inflating ventilation.

Technical validity and adverse events with both devices, closely related to ETI acquisition and performance, have been poorly reported [21, 25]. We found a better ETI technical validity and less adverse events with the AWS, although epiglottis luxation and AWS blade malposition were frequently reported for experts and novices, respectively. In a clinical study, ETT impingement onto the laryngeal structures has also been observed in 4 % of 320 ETI, but this could be easily managed by adjusting the AWS blade direction [24]. Unlike previous manikin and clinical studies [21–24], we observed two cases of esophageal intubation by novice operators with the AWS. Numerous adverse technical events, a higher Cormack and Lehane grade and delayed DLM-ETI were expected with the DLM in inexperienced sub-groups and reflected the difficulty to acquire the DLM-ETI technique [4–6]. Interestingly, the DLM-ETI failure rate was not negligible (2.5 %) for experienced operators with a significant amount of dental pressure. This could be related to manikin head rigidity, which can be more difficult to intubate than patients [21, 25]. Furthermore, dental pressure by the DLM could have been overestimated as it was subjectively evaluated and not based on the number of audible teeth clicks [21, 25] or video-recording. Indeed, a video-recording for each attempt would have been useful to improve attempts analysis and their technical validity as well as limit the potential subjective interpretation of investigators, especially for DLM-ETI. Furthermore, watching video-recordings would have allowed to operators to see and understand their potential mistakes and therefore to improve themselves. However, such video-recordings would have been very time-consuming for all the 1200 ETI attempts.

The simplicity and facility of AWS use were confirmed by an ETI qualitative evaluation by all operators including global satisfaction and preference. Nevertheless, AWS assembly and ETT manipulation were considered to be slightly more difficult by expert and novice 1 sub-groups. One explanation could be that AWS assembling can be less intuitive as the blade requires to be attached and locked to the monitor by a metallic collar and clip, and the ETT must be maintained in place while the AWS blade is removed laterally [18]. It is noteworthy that all operators considered that the DLM-ETI required more force of traction than did the AWS-ETI. This finding could explain, in part, the significant frequency of excessive dental pressure reported with the DLM including expert operators, as well as the higher ETI failure rate and longer ETI time for novices. Our findings on global satisfaction for the AWS confirm those of previous studies in inexperienced operators [7, 26, 27]. They also showed that DLM-ETI experts may rapidly appropriate the AWS-ETI technique.

AWS-ETI performance and easiness could probably be explained by its technical features. First, the disposable AWS blade is anatomically designed to conform to the shape of the mouth and pharynx and to pass over the tongue dorsum, with the minimal displacement of soft tissues. Second, unlike the DLM, the AWS does not require alignment of the oral, pharyngeal and tracheal axes to visualize the vocal cords [18]. Third, once inserted into the mouth, the AWS requires minimal adjustment of its position, whereas the DLM requires coordinated movements to expose the glottis [21, 24]. Fourth, imposed by the anatomic shape of the blade, epiglottis loading with the AWS blade tip allows to get the best glottis view to perform reliable ETI. By contrast, the DLM-ETI does not require epiglottis loading, but the blade tip must be placed into the glossoepiglottic fold in order to tract epiglottis and getting direct view of the glottis space [28]. This technical features, notably the need for loading the epiglottis or not, could explain in part the difference between both devices regarding ETI performance such as handling and the risk of adverse events or injuries. Therefore, the entire ETI procedure being performed under visual control with the AWS monitor, ETI could be more secured and supervised.

Simulation program and manikin studies are now essential for operator’s skill acquisition of life-saving procedures like ETI in the ICU environment [29–31]. Anyway, clinical studies are obviously needed. A recent meta-analysis, involving nine clinical ICU studies, demonstrated that VDL-ETI in ICU could be useful to decrease difficult ETI, esophageal intubation, Cormack 3/4 grades, and increase first-attempt success, but did not reduce severe complications [16]. However, these results should be interpreted with caution due to the between study heterogeneity depending on the outcome analyzed, operator experience and different VDL devices used. In fact, these ICU studies primarily assessed the GlideScope (GVL^®^; Verathon Medical, Bothell, WA, USA) [16], a VDL with a different conception and operating mode, mainly due to a deported screen and the use of a separate preformed metal stylet inserted into the ETT. In contrast, two prospective randomized clinical cohort studies performed by inexperienced providers outside the ICU found better performances for the AWS [22, 23]. Furthermore, due to their technical conception, the best glottis view provided by different VDL may not always match ETI outcomes [9, 13]. AWS advantages result from its integrated monitor and ETT side channel into the handle and blade, respectively, allowing to facilitate the ETT insertion and to visualize its entire progression [24]. These features are useful to avoid the difficulties due to the necessary coordination between a separate monitor and/or the use of an additional stylet. In addition, other VDL may partially blind the ETT progression and result in serious complications [32]. It must be underlined that our experimental manikin study has been conducted with a non-difficult airway head, and results cannot be expanded in cases of difficult ETI. Experimental and clinical studies have previously shown, however, that AWS-ETI could be a reliable technique in different difficult airways such as pharyngeal obstruction, cervical spine rigidity, tongue edema [26, 33] and limited mouth opening (≈20 mm) [34]. AWS can also be used according to a multimodal approach combining a flexible stylet or a fiberoptic bronchoscope, particularly if nasal ETI is required [35].

Nevertheless, there are some situations at risk of AWS-ETI difficulty or failure. The blade introduction in the mouth can be difficult due to the device length. However, the AWS blade introduction first, and secondarily connect to the AWS handle, may permit to introduce the entire device in the mouth. Despite a specific blade-side channel for introducing an aspiration catheter, abundant pharyngeal secretions can also obstruct the LCD glottis view, as well as condensation on the blade extremity [24]. In rare cases, the larynx can be difficult to reach due to a shorter length of the blade [36]. Finally, the minimum mouth opening required for the AWS-VDL insertion appears to be 20–25 mm [34], and AWS usefulness in cases of sub-glottic tumors needs to be assessed, likely in association with fiberoptic bronchoscope [37].

Therefore, all these features are strong arguments to initially perform manikin studies and to conduct then prospective clinical studies with each type of VDL, in no difficult as well as difficult ETI settings, before to determine the VDL-ETI’s place in the ICU [38].

Some limitations must be underlined in our study. First, it was a manikin study that may not strictly reproduce the human clinical conditions for ETI, particularly in the ICU regarding the underlying disease and technical circumstances. However, the manikin’s airway has been recognized as an acceptable and realistic condition for ETI evaluation [29–31]. Second, as it was obviously not possible to perform a blind study, a potential bias may exist, particularly for experts and investigators. Nevertheless, this bias was probably reduced, as our main endpoints were clearly defined. Lastly, we used subjective qualitative criteria as secondary endpoints for which the evaluation may widely change. However, this was probably unlikely as we found a good agreement between these criteria and our objective endpoints, particularly for ETI success and time.

## Conclusions

This large experimental manikin controlled study demonstrates that the AWS allowed faster, easier and more reliable ETI than did the DLM whatever the previous airway ETI experience. It also confirms that DLM-ETI needs a prolonged learning, whereas AWS-ETI requires less operator skill than does the DLM to effectively and rapidly secure the airway. Our results further suggest that the AWS may be a useful device for ETI learning with the DLM. Nevertheless, further randomized clinical studies are still warranted to determine the respective place of different VDL devices for ETI practice, particularly in the ICU setting.

## Abbreviations

AWS: Airway Scope
AWS-ETI: Endotracheal intubation with Airway Scope
AWS-VDL: Airway Scope video-laryngoscope
DLM: Direct laryngoscopy with Macintosh
DLM-ETI: Endotracheal intubation with Macintosh laryngoscope
ETI: Endotracheal intubation
ETT: Endotracheal tube
ICU: Intensive care unit
VDL: Video-laryngoscope
VDL-ETI: Endotracheal intubation with video-laryngoscope
